# Supplementary material for: Network structure of brain atrophy in de novo Parkinson's disease
Source: eLife. 2015 Sep 7;4:e08440. doi: 10.7554/eLife.08440 (PMC4596689; doi:10.7554/eLife.08440)
Supplement: Figure 4—source data 1. — DOI: http://dx.doi.org/10.7554/eLife.08440.016 [file elife08440s001.docx]

| Brain Region | Brain Structure | Side | Coordinates (center of mass) | | |
| --- | --- | --- | --- | --- | --- |
|  |  |  | x | y | z |
| supratentorial (Hammers atlas) | Hippocampus | R/L | 28/-28 | -16/-18 | -19/-18 |
|  | Amygdala | R/L | 23/-24 | -4/-5 | -21/-21 |
|  | Anterior temporal lobe (medial part) | R/L | 30/-31 | 9/8 | -37/-38 |
|  | Anterior temporal lobe (lateral part) | R/L | 49/-50 | 8/7 | -36/-36 |
|  | Parahippocampal gyrus | R/L | 24/-25 | -17/-17 | -27/-27 |
|  | Superior temporal gyrus (posterior part) | R/L | 53/-53 | -14/-14 | -2/-3 |
|  | Middle and inferior temporal gyrus | R/L | 55/-56 | -15/-16 | -25/-24 |
|  | Fusiform gyrus | R/L | 35/-36 | -15/-16 | -34/-33 |
|  | Insula | L/R | -34/34 | -2/0 | 1/0 |
|  | Occipital lobe (lateral part) | L/R | -30/-31 | -81/-80 | 10/10 |
|  | Anterior cingulate gyrus | L/R | -5/6 | 30/25 | 23/26 |
|  | Posterior cingulate gyrus | L/R | -5/5 | -28/-27 | 36/36 |
|  | Middle frontal gyrus | L/R | -32/33 | 30/32 | 37/26 |
|  | Posterior temporal lobe | L/R | -46/47 | -47/-46 | -3/-2 |
|  | Parietal lobe (Inferiolateral) | L/R | -48/48 | -45/-43 | 34/36 |
|  | Caudate nucleus | L/R | -10/11 | 10/11 | 10/10 |
|  | Nucleus accumbens | L/R | -10/8 | 9/9 | -9/-9 |
|  | Putamen | L/R | -26/26 | 2/3 | 0/0 |
|  | Thalamus | L/R | -13/12 | -19/-19 | 6/6 |
|  | Pallidum | L/R | -20/20 | -2/-2 | -2/-2 |
|  | Precentral gyrus | L/R | -33/34 | -9/-8 | 45/45 |
|  | Straight gyrus | L/R | -6/4 | 28/29 | -21/-21 |
|  | Anterior orbital gyrus | L/R | -24/24 | 50/50 | 14/-15 |
|  | Inferior frontal gyrus | L/R | -46/48 | 24/23 | 9/9 |
|  | Superior frontal gyrus | L/R | -12/13 | 31/32 | 39/38 |
|  | Postcentral gyrus | L/R | -37/37 | -21/-22 | 44/44 |
|  | Superior parietal gyrus | L/R | -18/18 | -53/-53 | 47/47 |
|  | Lingual gyrus | L/R | -14/13 | -71/-71 | -4/-2 |
|  | Cuneus | L/R | -8/9 | -81/-78 | 18/20 |
|  | Medial orbital gyrus | L/R | -11/10 | 41/42 | -20/-20 |
|  | Lateral orbital gyrus | L/R | -40/42 | 40/41 | -14/-12 |
|  | Posterior orbital gyrus | L/R | -28/27 | 23/24 | -18/-17 |
|  | Subgenual frontal cortex | L/R | -7/5 | 25/25 | -10/-9 |
|  | Subcallosal area | L/R | -5/3 | 12/12 | -8/-7 |
|  | Pre-subgenual frontal cortex | L/R | -5/4 | 39/37 | -6/-6 |
|  | Superior temporal gyrus (anterior part) | L/R | -49/50 | 13/13 | -21/-20 |
| Cerebellum (SUIT atlas) | lobule I and IV | L/R | -7/9 | -46/-45 | -16/-17 |
|  | lobule V | L/R | -14/14 | -51/-53 | -18/-18 |
|  | lobule VI | L/Vermis/R | -23/24/1 | -59/-60/-71 | -25/-25/-22 |
|  | Crus I | L/Vermis/R | -35/36/-4 | -68/-69/-78 | -34/-33/-28 |
|  | Crus II | L/Vermis/R | -25/26/1 | -74/-75/-75 | -44/-43/-33 |
|  | lobule VIIb | L/Vermis/R | -25/27/1 | -65/-66/-70 | -52/-52/-33 |
|  | lobule VIIIa | L/Vermis/R | -23/25/1 | -58/-59/-68 | -54/-54/-39 |
|  | lobule VIIIb | L/Vermis/R | -16/18/1 | -51/-52/-64 | -55/-55/-42 |
|  | lobule IX | L/Vermis/R | -6/7/1 | -54/-54/-57 | -49/-49/-38 |
|  | lobule X | L/Vermis/R | -20/22/1 | -39/-40/-50 | -44/-45/-35 |
|  | Dentate nucleus | L/R | -14/16 | -35/-35 | -35/-35 |
|  | Interposed nucleus | L/R | -6/8 | -59/-58 | -30/-29 |
|  | Fastigial nucleus | L/R | -1/4 | -55/-55 | -28/-27 |
| Brainstem | Red nucleus | L/R | -6/5 | -19/-19 | -9/-8 |
|  | Substantia nigra | L/R | -11/10 | -17/-17 | -12/-12 |
|  | Subthalamic nucleus | L/R | -12/13 | -13/-14 | -6/-6 |
