# Supplementary material for: Network structure of brain atrophy in de novo Parkinson's disease
Source: eLife. 2015 Sep 7;4:e08440. doi: 10.7554/eLife.08440 (PMC4596689; doi:10.7554/eLife.08440)
Supplement: Figure 4—source data 2. — Each brain region from the atlas was used as a potential propagator. The statistical difference (t-value) between the average deformation in PD and controls in each region was used as an atrophy measure. The correlation between this atrophy measure and the anatomical (or geodesic) distance to the potential propagator was used as a measure of propagation strength. The potential propagator regions are sorted by correlation values. DOI: http://dx.doi.org/10.7554/eLife.08440.017 [file elife08440s002.docx]

| Seed region | r | Seed region | r |
| --- | --- | --- | --- |
| Cerebellum VIIb | -0.28 | Subthalamic Nucleus | -0.11 |
| Substantia Nigra | -0.28 | Cuneus | -0.10 |
| Cerebellum X | -0.28 | Red Nucleus | -0.09 |
| Cerebellum VIIIa | -0.27 | Hippocampus | -0.09 |
| Cerebellum VIIIb | -0.27 | Anterior temporal lobe (lateral part) | -0.08 |
| Cerebellum Vermis VIIb | -0.27 | Superior temporal gyrus (posterior part) | -0.06 |
| Cerebellum CrusII | -0.26 | Parietal lobe (Inferiolateral) | -0.05 |
| Cerebellum Vermis VIIIa | -0.25 | Superior temporal gyrus (anterior part) | -0.01 |
| Cerebellum Vermis CrusII | -0.25 | Thalamus | 0.02 |
| Cerebellum CrusI | -0.25 | Superior parietal gyrus | 0.03 |
| Cerebellum Vermis VIIIb | -0.24 | Pallidum | 0.03 |
| Cerebellum Vermis CrusI | -0.24 | Insula | 0.12 |
| Cerebellum IX | -0.23 | Putamen | 0.14 |
| Cerebellum VI | -0.23 | Caudate nucleus | 0.17 |
| Cerebellum Vermis IX | -0.23 | Postcentral gyrus | 0.18 |
| Cerebellum Vermis VI | -0.22 | Nucleus accumbens | 0.20 |
| Cerebellum Dentate | -0.21 | Subcallosal area | 0.23 |
| Cerebellum Interposed | -0.20 | Posterior orbital gyrus | 0.23 |
| Cerebellum Fastigial | -0.19 | Posterior cingulate gyrus | 0.24 |
| Cerebellum V | -0.18 | Medial orbital gyrus | 0.27 |
| Parahippocampal gyrus | -0.18 | Straight gyrus | 0.29 |
| Cerebellum I IV | -0.18 | Subgenual frontal cortex | 0.29 |
| Cerebellum Vermis X | -0.18 | Lateral orbital gyrus | 0.31 |
| Middle and inferior temporal gyrus | -0.15 | Precentral gyrus | 0.32 |
| Occipital lobe (lateral part) | -0.14 | Inferior frontal gyrus | 0.32 |
| Lingual gyrus | -0.14 | Anterior orbital gyrus | 0.33 |
| occipitotemporal gyrus (lateral part) | -0.13 | Superior frontal gyrus | 0.35 |
| Posterior temporal lobe | -0.13 | Anterior cingulate gyrus | 0.35 |
| Amygdala | -0.13 | Middle frontal gyrus | 0.35 |
| Anterior temporal lobe (medial part) | -0.12 | Pre-subgenual frontal cortex | 0.36 |
